# Editorial: Pharmacological Aspects of Ligand-Gated Ion Channels as Targets of Natural and Synthetic Agents

**DOI:** 10.3389/fnins.2022.895299

**Published:** 2022-04-08

**Authors:** César Mattei, Hélène Tricoire-Leignel, Christian Legros, Richard J. Lewis, Jordi Molgó

**Affiliations:** ^1^Univ Angers, INSERM, CNRS, MITOVASC, Equipe CarMe, SFR ICAT, Angers, France; ^2^Centre for Pain Research, Institute for Molecular Bioscience, The University of Queensland, St. Lucia, QLD, Australia; ^3^CEA (French Alternative Energies and Atomic Energy Commission), INRAE, University of Paris-Saclay, DMTS, SIMoS, ERL CNRS 9004, Gif sur Yvette, France

**Keywords:** LGICs, GABA_A_R, nAChR, NMDA GluR, P2XR, toxins, natural ligands, synthetic molecules

Ligand-gated ion channels (LGICs) are ubiquitously expressed in all biological systems. In addition to participating in the ionic fluidity of living membranes, they are at the origin of the intracellular transduction of messages from the extracellular medium. They are considered as proven or potential therapeutic targets, as many drugs enhance, inhibit, or modulate their activity, and thus disrupt the cell biological response in tissues affected by pathophysiological dysfunctions.

In this special issue, several papers focus on the functions of LGICs as targets of exogenous ligands including neurotoxic agents. The aim of this collection is to improve our understanding of the versatile pharmacology of LGICs at multiple scales: *in vivo, in vitro* and *in silico*.

Given their importance in inhibitory brain networks, GABA_A_ receptors are one of the most studied LGICs. The recent elucidation of their structure using high-resolution cryo-electron microscopy constituted a breakthrough for the dissection of their pharmacological interactions at the molecular level (Zhu et al., 2018; Laverty et al., 2019). Singh and Villoutreix took advantage of these knowledges to investigate the binding of pyrazoloquinolinones, a group of non-sedative anxiolytics, to the α1β3γ2 receptor, highlighting the implication of several residues at the α1+/β3- interface. The same α1β3γ2 receptor is shown by Benkherouf et al. to be *in vitro* targeted by humulone, an active metabolite of hops. Humulone acts as a positive allosteric modulator and displays both sedative and hypnotic properties *in vivo* (Benkherouf et al.). As its effects are potentiated by ethanol, it raises the question of intoxicating effects on the central nervous system of ethanol in hops-containing alcohols.

The subunit composition of GABA_A_ receptors−2α, 2β and a third one—is a key factor in their pharmacological properties, as some therapeutic or toxic compounds exert their effects preferentially on certain receptor conformations and less on others. Thus, using an *in vitro* approach on Xenopus oocytes Richeter et al. show the importance of the third subunit in the activity of non-benzodiazepine Z-compounds on the GABA current using concatenated receptors. These data provide insight into the difference in activity between these Z-compounds and the benzodiazepine anxiolytics. A similar approach was used by Soualah et al. who reveal a major role for the third subunit in the antagonist effects of fipronil, an insecticide targeting these receptors. Characterizing the activity of a ligand on GABA_A_Rs seems accessible using the pyrazoloquinolinone scaffold in such a way as to exhaustively anticipate the molecular interaction between an active molecule and these receptors, given the plurality of possible binding sites. This is what is proposed by Fabjan et al. in an elegant structure activity relationship by computational methods and docking modeling. The challenge of synthetic molecule selectivity is also addressed by Golani et al. who show, *in vitro* (binding tests and cellular electrophysiology), *in vivo* (anxiolysis and sedation) and *in silico* (docking modeling), the importance of subunits as therapeutic targets of sedative or anxiolytic agents, using analogs of triazolam. Finally, Castellano et al. review the recent developments on the pharmacology of GABA_A_Rs, from a historical and scientific perspective. Indeed, several accessory proteins have been recently identified to influence the activity of these receptors (Castellano et al.).

In addition to GABA_A_Rs, nicotinic ACh receptors (nAChRs) are cationic channels that are widely expressed in the CNS and PNS. They are structurally similar to GABA_A_Rs. Ho et al. propose an exhaustive review of neurotoxins of plant, animal and microalgal origin, showing the structure-function relationship of these toxins. Beyond their toxicological aspect, they represent an immense reservoir of pharmacological tools to characterize the functioning of nAChRs (Ho et al.). Because they are also present in the nervous system of invertebrates, nAChRs have long been a target for natural or synthetic pesticides. Kaji et al. offer the first pharmacological characterization of human hookworm nAChRs, by cloning and expressing the ACR-16 subunit of the nematode parasites *Ancylostoma ceylanicum* and *Necator americanus*. On this subject of antiparasitic molecules, Castro et al. show, by an approach combining *in vivo* toxicology on *C. elegans* and electrophysiology on Xenopus oocytes, that doxepinone acts as an antagonist of Glutamate-Activated Chloride Channels. These anionic glutamatergic receptors are of particular interest as they are not expressed in the vertebrate nervous system.

Ionotropic glutamatergic receptors are also an important target for therapeutic agents and toxic molecules due to their involvement in brain pathologies. Activation of N-methyl-D-aspartate receptors (NMDARs) is a pivotal mechanism of cerebral ischaemia, making these receptors a key target. Liu et al.used the phytoestrogen icaritine (ICT) as a neuroprotective agent *in vitro* after showing its beneficial activity *in vivo*. ICT reduces cell death in glutamate-challenged rat cortical neurons, *via* activation of the intracellular ERK pathway and modification of the NMDARs subunit expression (Liu et al.). NMDARs are furthermore central to the study of Sun et al. which shows that a chronic repeated restraint stress induces cognitive impairments in juvenile male rats. This was correlated with a reduced density of dendritic spines, and consecutively an enhanced long-term depression and reduced long-term potentiation in the hippocampus. NMDAR subunit expression was increased in the CA1 region of the hippocampus (Sun et al.). Horak et al. review the specific place of extracellular domains of NMDAR subunits in endoplasmic reticulum processing and quality control. Finally, NMDARs have been shown to be a target of interest in the pharmacological management of schizophrenia. Wu et al. provide a comprehensive review of the mechanisms underlying the deficits observed in schizophrenia, the role of NMDARs, and the molecules promoting their activity and tested on humans.

Finally, several experimental studies have focused on other LGICs, particularly in the area of pain: TRPV1, ASIC and P2X4 receptors. Given its involvement in the transduction of hot pain and inflammatory hyperalgesia, the TRPV1 channel, a polymodal receptor for vallinoid and endovallinoid compounds, is now considered a major therapeutic target for the management of various forms of chronic pain. Vitamin B2 (also known as riboflavin) is a dietary molecule that has antagonistic effect on capsaicin-activated TRPV1, as shown by Lee et al., who combine *in vivo* itch model with *in vitro* electrophysiology on sensory neurons and HEK cells. In neuropathic pain phenomena, P2X4 purinergic receptors are overexpressed and favors the pain pathway to the CNS. Wang et al. took advantage of the vegetal astragalin to manage neuropathic pain *in vivo* using a model of nervous constriction injury. Astragalin acts both by decreasing P2X4R expression and glial activation *in vivo* and by reducing ATP-induced purinergic current *in vitro*. Finally, the interplay between ASICs channels and α2A adrenergic receptors (α2-ARs) was investigated in the pain establishment by tissue acidosis (Wei et al.). Selective activation of α2-ARs with dexmedetomidine decreases the amplitude of ASIC currents in DRG neurons, and relieves pain induced by intraplantar acid injection in rats.

Pain, stress, anxiety, ischaemia, motor skills... LGICs are linked to many functions. Therefore, we believe that a better understanding of their pharmacology can contribute to the knowledge of their implication in deleterious or virtuous processes. This topical collection participates to achieve this goal, and the published papers add to a comprehensive view of the full potential of this knowledge.

## Author Contributions

CM wrote the editorial. HT-L, CL, RL, and JM revised the work. All authors contributed to the article and approved the submitted version.

## Conflict of Interest

The authors declare that the research was conducted in the absence of any commercial or financial relationships that could be construed as a potential conflict of interest.

## Publisher's Note

All claims expressed in this article are solely those of the authors and do not necessarily represent those of their affiliated organizations, or those of the publisher, the editors and the reviewers. Any product that may be evaluated in this article, or claim that may be made by its manufacturer, is not guaranteed or endorsed by the publisher.
